# Exploring the impact of verbal encouragement on strength, endurance, and psychophysiological responses: enhancing teaching strategies in sports science education

**DOI:** 10.3389/fspor.2024.1360717

**Published:** 2024-07-10

**Authors:** Amir Romdhani, Faten Sahli, Hatem Ghouili, Omar Trabelsi, Mahmoud Rebhi, Mohamed Ben Aissa, Mouna Saidane, Noomen Guelmami, Ismail Dergaa, Monoem Haddad, Makram Zghibi

**Affiliations:** ^1^ High Institute of Sport and Physical Education of Ksar Said, University of Manouba, Manouba, Tunisia; ^2^ High Institute of Sport and Physical Education of Kef, University of Jendouba, Kef, Tunisia; ^3^ High Institute of Sport and Physical Education of Sfax, University of Sfax, Sfax, Tunisia; ^4^ High School of Nursing Sciences, University of Jendouba, Kef, Tunisia; ^5^ Postgraduate School of Public Health, Department of Health Sciences (DISSAL), University of Genoa, Genoa, Italy; ^6^ Primary Health Care Corporation (PHCC), Doha, Qatar; ^7^ Physical Education Department, College of Education, Qatar University, Doha, Qatar

**Keywords:** verbal support, teaching strategies, physical activity, enjoyment, perceived exertion

## Abstract

**Introduction:**

This study investigates the effects of teacher verbal encouragement (VE) on strength, endurance, and psychophysiological responses, aiming to enhance teaching strategies in sports science education.

**Methods:**

Forty-eight sports science students, aged 21.3 ± 0.5 years, participated in this study. The sample was randomly divided into two groups, and a counterbalancing procedure was implemented. Participants completed strength and endurance testing sessions under normal conditions in the first week and repeated similar sessions in the second week with teacher VE. Strength was assessed using the 1RM bench press, squat, and deadlift tests, while endurance was evaluated through 8-minute time trials (8MTT: burpees, box jumps, hand-release push-ups, and sit-ups). Perceived exertion and physical activity enjoyment were investigated using self-reporting instruments.

**Results:**

The key findings showed that participants lifted greater weights in the 1RM bench press (*p* < 0.01; *r**** ***= 0.45, medium to large effect), squat (*p* < 0.001; Hedges' *g* = 1.36, large effect), and deadlift tests (*p* < 0.001; *r *= 0.79, large effect) and completed a greater number of repetitions in the 8MTT (*p* < 0.001; *r *= 0.87, large effect) under VE. Perceived exertion was found to be lower under normal conditions (*p* < 0.05; *r *= 0.29, small effect), yet physical activity enjoyment significantly increased under VE (*p* < 0.05; *r *= 0.81, large effect).

**Discussion:**

In conclusion, implementing teacher VE in sports science education can contribute to improved strength and endurance training outcomes and student psychophysiological response.

## 1 Introduction

Extensive research has been conducted on the effects of verbal encouragement (VE) on sports performance (1–3) and motor learning in physical education (PE) (4, 5). A multitude of studies have investigated the influence of VE on various facets, encompassing physiological parameters (6), psycho-physiological responses (7, 8), and motor performance (9). The scientific literature consistently affirms that VE is a highly effective method for boosting motor and physical performance (10).

In a study by Andreacci et al. (11), it was observed that consistent and repeated VE during a treadmill test (specifically, every 20 s and 60 s) resulted in significantly higher levels of maximum effort compared to scenarios where no VE is provided or when it is given infrequently (e.g., every 180 s). Furthermore, the findings from the study conducted by Amagliani et al. (12) suggest that, regardless of their training background, college-aged women generated the highest level of force during a maximal voluntary isometric contraction when both verbal and visual encouragement were provided. Their results highlight the importance of incorporating both VE and visual feedback as key factors in eliciting peak force production (12). More interestingly, the findings of Innes et al. (13) emphasize the importance of incorporating supportive peer environments and VE strategies in promoting positive self-efficacy beliefs and enhancing performance outcomes in school-aged children. More interestingly, Hammami et al. (14) conducted a comprehensive study focusing on the advantageous effects of combining VE with favorite music as a means to enhance conditioning training. The incorporation of VE, delivered through motivating words and cues, alongside the accompaniment of the music preferred by the 17-year-old participating basketballers, proved to be a powerful strategy in boosting performance in the repeated change-of-direction test (14).

The impact of VE extends beyond the individual athlete to team dynamics (15–17). Effective communication through positive VE fosters a supportive team environment, enhancing collaboration, cohesion, and overall team performance (15). Coaches and teammates who provide constructive and motivating feedback can inspire and uplift athletes, creating a culture of encouragement and continuous improvement (8, 15).

The literature surrounding VE and its effects on sports performance is extensive and encompasses various sporting disciplines. Researchers have consistently investigated the role of VE as a motivational pedagogical tool to enhance athlete and student performance in various sports such as soccer (2, 7, 8, 17), basketball (14), handball (16), swimming (3), recreational youth sports (18), and running events (1). Muscular strength assumes a substantial role in sports performance, and previous studies have substantiated its susceptibility to various factors, including VE (12, 19). This latter serves as a widely employed motivational tool, particularly in high-intensity exercise scenarios (6, 19). In a investigation by Engel et al. (19), beneficial effects were observed in functional strength tests, particularly at high intensity levels. The study explored the positive impact of VE on both strength and endurance among experienced High-Intensity Functional Training (HIFT) athletes. The authors also investigated whether the VE provided by coaches influenced the reliability of the tests. Their findings demonstrated constructive effects on specific strength and endurance within the HIFT context, alongside improvements in the reliability measures during the testing process. Similarly, a study conducted by Belkhiria et al. (20) examined the effects of VE on isometric strength and electromyographic parameters. This investigation involved 23 subjects performing a grasping task, and the results substantiated the significant influence of VE on isometric strength exercises.

Studies have also revealed that VE has a positive influence on athletes' psychological state, boosting their self-confidence, motivation, and focus during training and competitions (5, 7, 8). Reportedly, athletes who receive consistent and specific VE from coaches and teammates tend to exhibit higher levels of effort, persistence, and determination, leading to improved performance outcomes (13, 15). Moreover, the use of VE has been shown to positively impact physiological responses during exercise. When athletes receive encouraging words, their perception of effort may decrease, allowing them to push themselves further and achieve higher levels of performance (8). This can be particularly beneficial in high-intensity activities where fatigue and mental barriers often play a significant role (14, 21). It is worth noting that the effectiveness of VE may vary depending on individual athletes' personalities, preferences, and the specific sport or activity (22). Some athletes may respond more positively to direct, authoritative encouragement, while others may thrive with more nuanced, emotionally supportive approaches (23, 24).

Consistently, research reveals the profound impact that VE wields as a powerful pedagogical tool in different educational contexts. Tuckman and Sexton (25) shed light on its vital role in nurturing the fragile self-esteem of students, fortifying their confidence, and fostering academic achievement. More interestingly, Brown and Howard (26) found that using verbal encouragement, especially through a Socially Interactive Robotic Tutor (SIRT), boosts student engagement during math learning. Results from 44 students showed verbal cues significantly increased engagement compared to non-interactive methods, regardless of age or math level. The study of Guéguen et al. (27) also found that VE significantly improved children's performance across tasks, with 77% of 3–5-year-olds and 82% of 8–9 year-olds succeeding when given VE, compared to lower success rates in control conditions. This underscores the importance of utilizing VE in teaching practices.

Given the undeniable significance of understanding how VE impacts crucial physical parameters such as strength and endurance, as well as its potential as a pedagogical tool in educational contexts, further research on its impact within sports science education remains of utmost importance. Consequently, the present study investigates the effects of VE by conducting rigorous investigations into strength and endurance testing within authentic sports science education environments. In addition, the study aims to explore the intricate interplay of psychophysiological and physiological responses that accompany VE. By unraveling these intricacies, this research seeks to contribute valuable insights that can inform and enhance teaching strategies in sports science programs, optimize athletic training programs, and ultimately elevate sports performance to new heights. The rationale for this study stems from the need to investigate the effects of VE specifically within the context of sports science education. While previous research has examined the impact of VE on professional athletes across various age groups, or on physical education students at lower educational levels such as primary, middle, and secondary school, there is a gap in understanding its effects on sports science students. We hypothesized that the provision of consistent and repeated VE by the teacher during strength and endurance tests would result in significant improvements in performance, perceived exertion, and psychological responses in sports science students.

## 2 Materials and methods

### 2.1 Participants

A total of 48 final-year undergraduate sports science students from the High Institute of Sport and Physical Education of Kef (Kef, Tunisia), comprising 24 males and 24 females, with an average age of 21.3 ± 0.5 years, willingly volunteered to take part in this study. By including an equal number of males and females, we aimed to minimize the potential confounding effects of gender, allowing for a more accurate assessment of the variables under investigation. The male participants had an average body mass of 70.2 ± 9.3 kg, a body height of 175.1 ± 10.2 cm, and a body mass index (BMI) of 22.7 ± 2.6 kg/m^2^. On the other hand, the female participants had an average body mass of 60.5 ± 8.4 kg, a body height of 165.3 ± 8.7 cm, and a BMI of 21.3 ± 1.9 kg/m^2^.

In order to calculate the optimal sample size for our study, we used the GPower 3.1 software (28). With a significance level (*α*) set at 0.05 and a medium effect size (Cohen's d) of 0.5, our calculations indicated that a sample size of 44 participants would grant us 90% statistical power to effectively identify significant and meaningful outcomes. To ensure the robustness of our findings and account for any potential dropouts or unforeseen circumstances, we decided to recruit a slightly larger number of participants. As a result, a total of 48 participants were included in our study, exceeding the required sample size.

The participants included in this study were selected based on specific inclusion criteria. Firstly, they were chosen for their level of physical activity, engaging in regular physical activity for approximately 6 h per week. Additionally, the participants possessed prior experience in relevant activities, ranging from 2 to 4 years. To ensure the validity and reliability of the study, all participants underwent medical examinations, including assessments of cardiovascular health through measures such as resting heart rate, blood pressure, and electrocardiogram (ECG) analysis. Musculoskeletal integrity was evaluated using techniques such as joint range of motion assessments and orthopedic examinations to detect any existing injuries or limitations. None of the participants reported having any neuromuscular or cardiovascular disorders, nor did they have any specific musculoskeletal injuries affecting the ankles, knees, or hips. This exclusion criterion was crucial to ensure that the participants were free from any conditions that could potentially confound the results or compromise their ability to perform the required physical tasks accurately.

### 2.2 Ethical considerations

Prior to their participation, all individuals involved in the study provided written informed consent. They were provided with detailed verbal and written explanations regarding the experimental design and potential risks associated with their involvement. The study was conducted in strict adherence to the principles outlined in the most recent version of the Helsinki Declaration, ensuring the ethical considerations and welfare of the participants. The research protocol underwent a thorough review and received full approval from the Ethics Committee of the High Institute of Sports and Physical Education of Kef, University of Jendouba (0040-2023).

### 2.3 Study design

The present study employed a randomized crossover design, wherein all participants completed the strength and endurance test under both VE and normal conditions (without VE). The washout period between testing procedures ranged from 72 to 96 h, as detailed in Table 1. The 48 participants were randomly divided into two groups (Group 1 and Group 2), ensuring equal distribution based on both number and sex. Both groups were exposed to both conditions; however, the order of exposure was reversed between the groups. The primary objective of this procedure was to minimize potential nuisance effects, including order of exposure (carryover effect) and time of day variations.

**Table 1:** Counterbalancing procedure explained: controlling systematic biases.

Week	Session	Group	Condition		Time of day		Tests			
			Without VE	With VE	9:00 a.m.	2:00 p.m.	1RM	8MTT	RPE	PACES
1	1 (Mon.)	1	**×**	** **	**×**	** **	**×**	** **	** **	** **
		2	** **	**×**	** **	**×**	** **	**×**	**×**	**×**
	2 (Thurs.)	1	**×**	** **	**×**	** **	** **	**×**	**×**	**×**
		2	** **	**×**	** **	**×**	**×**	** **	** **	** **
2	3 (Mon.)	1	** **	**×**	** **	**×**	** **	**×**	**×**	**×**
		2	**×**	** **	**×**	** **	**×**	** **	** **	** **
	4 (Thurs.)	1	** **	**×**	** **	**×**	**×**	** **	** **	** **
		2	**×**	** **	**×**	** **	** **	**×**	**×**	**×**

Mond., Monday; Thurs., Thursday; VE, verbal encouragement; 1RM, one-rep maximum assessments; 8MTT, 8-min time trials; RPE, rating of perceived exertion; PACES, physical activity enjoyment scale.

During each of the two experimental weeks, each group participated in a designated strength testing session and an endurance testing session. In the first week, if a group completed both sessions under VE, they repeated the same sessions in the second week under normal conditions (without VE), and vice versa for the other group. Furthermore, if the sessions were conducted at 9 a.m. during (morning period) in the first week, they were rescheduled for 2 p.m. (afternoon period) in the second week, and vice versa for the other group. Detailed information regarding the counterbalancing procedure can be found in Table 1.

### 2.4 Study procedures

#### 2.4.1 Familiarization session

To ensure that participants were well-prepared and confident in the testing procedures, a comprehensive familiarization session was conducted one week before the main experiment. During the familiarization session, participants were given ample time to practice using the instruments and equipment that would be utilized during the study. Anthropometric measurements, such as height and weight, were taken at the beginning of the familiarization session. The familiarization session, guided by the same teacher involved in the main experimental procedures, lasted for 2 h and 30 min, commencing at 9 a.m. Throughout various parts of the session, the teacher VE expressions.

During this session, we utilized an estimation process to determine the starting weight for the one-repetition maximum (1RM) in squat, deadlift and bench press tasks of each participant. This process involved allowing the participants to perform several submaximal lifts with progressively increasing weights. Based on their performance and feedback, we were able to estimate an appropriate starting weight that would allow them to safely and effectively work towards their actual 1RM in experimental sessions. By using this approach, we aimed to ensure that the participants were adequately challenged while minimizing the risk of injury or excessive fatigue. Furthermore, by having an initial estimate of the participants' strength capabilities, we were able to avoid unnecessary attempts with non-challenging weights, which could potentially waste valuable time during experimental sessions.

#### 2.4.2 Study exposures

Overall, the study consisted of two experimental weeks during which participants completed two strength testing sessions and two endurance testing sessions (Table 1). Each participant completed one session under normal conditions (without VE) and another session with VE provided by their teacher. The testing sessions were carried out at the gym of the high institute of sport and physical education of Kef during the month of March. The ambient temperature within the gym ranged between 10 and 16 degrees Celsius, depending on the time of day. A standardized 10-minute warm-up was conducted before each session, consisting of dynamic stretching, cardiovascular exercises, and mobility drills to prepare the participants physically and mentally. This warm-up protocol aimed to increase blood flow, improve flexibility, and activate the muscles, ensuring optimal readiness for the subsequent testing.

The teacher provided VE to the participants in Tunisian dialect throughout the duration of each test. The expressions used were standardized and included the following phrases: “Well done!”, “Keep going”, “You can do it”, and “Don't give up” (interpretive translation1). The teacher also personalized the encouragement by addressing each student by their name (e.g., “You can do it, Rami” or “Don't give up, Rami”) one time per testing task. The teacher employed a loud, energetic, and enthusiastic tone while providing encouragement to the participating students. The loudness of the teacher's voice helped to ensure that the VE was clearly audible to all the students being tested.

A single teacher was recruited to instruct and provide encouragement to all the participating students to ensure consistency in the delivery of VE across all test sessions. By having a single teacher, the study aimed to minimize potential variations in the tone, style, and content of the VE provided.

### 2.5 Data collection

#### 2.5.1 One-rep maximum (1RM) assessments

During each strength testing session, participants engaged in 2 sets of the 1RM bench press, squat, and deadlift tests. The order of the tests was determined following the recommendations put forth by Simão et al. (29), emphasizing the precedence of exercises stimulating larger muscle groups before those stimulating smaller muscle groups (e.g., bench press prior to pec-deck fly or squat prior to leg extension). The rationale behind this sequencing stems from the notion that pre-fatiguing smaller muscle groups (e.g., triceps brachii, anterior deltoids) by single-joint exercises (e.g., triceps extension, shoulder flexion) may impede the larger muscle groups (e.g., pectoralis major) from achieving an effective overload during subsequent multi-joint exercises (e.g., bench press), potentially limiting their capacity to sustain the load and/or repetitions per set.

Standardized rest periods of 2 min between repetitions, 3 min between exercises, and 5 min between sets were implemented during the strength testing sessions (30). At the end of each session, the maximum weight lifted by each participant in each test was recorded. During the familiarization session, participants received detailed explanations and demonstrations on the correct technical execution of each lift.

#### 2.5.2 8-min time trials (8MTT)

During the endurance testing sessions, participants were tasked with completing as many rounds as possible of a specified set of exercises within an 8-minute time frame. The exercises included 6 repetitions of burpees, 6 repetitions of box jumps, 6 repetitions of hand-release push-ups, and 10 repetitions of sit-ups. All movements performed during the endurance testing adhered to the standardized range of motion prescribed by the High-Intensity Functional Training (HIFT) guidelines established by CrossFit Inc. in 2017. The total number of movements completed within the 8-minute time frame served as an indicator of participants' endurance capacity. It provided valuable insights into their ability to sustain effort and maintain performance over an extended period.

#### 2.5.3 Rating of perceived exertion (RPE)

Perceived exertion was examined immediately after the completion of the 8MTT using the Rating of Perceived Exertion (RPE) instrument, developed by Borg (31). An edited version of Borg's original scale (32) was used in this study, namely the category-ratio 10-point scale (CR10). This widely recognized version allowed participants to subjectively rate and communicate their perceived level of exertion experienced during the intense 8MTT on a 10-point scale ranging from “really easy” (1) to “maximal effort” (10). Facial emojis were also assigned to each anchor point on CR10 scale to facilitate the ability to accurately rate perceived exertion. Anchors of the CR10 scale were translated from English to Arabic (participants' mother tongue) by three expert English-Arabic interpreters.

Participants were advised to disregard any isolated factors such as leg pain or shortness of breath, and instead, to concentrate on their overall perception of exertion during the 8MTT. By focusing on the holistic experience of exertion, participants were able to provide a comprehensive assessment of their subjective exertional intensity and fatigue level during the activity. Participants were inputted their ratings directly on the tablet, using the TypeForm platfrom, ensuring accuracy and ease of response.

#### 2.5.4 Physical activity enjoyment scale (PACES)

The original 18-item Physical Activity Enjoyment Scale (PACES) (33) was employed to assess participants' enjoyment of the 8MTT they had just engaged in. Participants were asked to rate their current feelings about the activity using a 7-point bipolar rating scale. Eleven items on the PACES are reverse scored. Higher scores on the PACES indicated higher levels of enjoyment experienced during the 8MTT. The PACES was administered immediately after the RPE assessment, following the completion of the 8MTT. Participants completed the PACES in a pencil-paper format.

The PACES items were translated from English to Arabic, the participants' mother tongue, by three expert English-Arabic interpreters. These interpreters possessed a deep understanding of both languages and had expertise in accurately translating psychological measures. For each item in the translation process, conformity was coded as 1 when there was agreement among the three translations, while any disparities or inconsistencies were coded as 0. To assess the inter-translator agreement for the entire scale, the conformity of all items across the translations was examined. The inter-translator agreement was calculated to be 0.96, indicating a high level of agreement among the translators. The robust inter-translator agreement further supports the reliability and validity of the Arabic version of the PACES, ensuring that the translated items captured the intended meaning and maintained consistency across translations.

### 2.6 Data analyses

A pooled analysis was implemented in this study, which involved combining the data of both groups from a specific test (e.g., squat test, bench press test, etc.) under specific conditions (without or with VE), regardless of the session in which the test was conducted. This pooling of data ensured that each participant in the study experienced all conditions or treatments in a balanced and systematic manner. This approach allowed for a more accurate evaluation of the effects of the different conditions on the study outcomes and enhanced the validity and reliability of the statistical analyses (34, 35).

Paired comparisons were performed on the data obtained from the different tests and measures, and the choice between parametric and non-parametric tests was determined based on the assumption of normality. This latter was assessed using the Shapiro-Wilk test, with *p*-values less than 0.05 indicating a violation of the assumption. In cases where the normality assumption was violated, the Wilcoxon signed ranks test was used instead of the *t*-test for paired samples. The significance level for both tests was set at 0.05.

To quantify the effect size, Hedges' g was calculated for the *t*-test comparisons, while Pearson's r was used for the Wilcoxon signed ranks test. In the text, the data were presented as mean ± standard deviation (SE) for the *t*-test comparisons, whereas median and interquartile range (IQR) were used for the Wilcoxon signed ranks test. In the figures, the data were presented as mean ± standard error (SE), regardless of the test used. All statistical analyses were conducted using SPSS software (Version 27, IBM Corp., Armonk, NY, USA).

## 3 Results

### 3.1 One-rep maximum (1RM) assessments

#### 3.1.1 Bench press test

**Figure 1:**
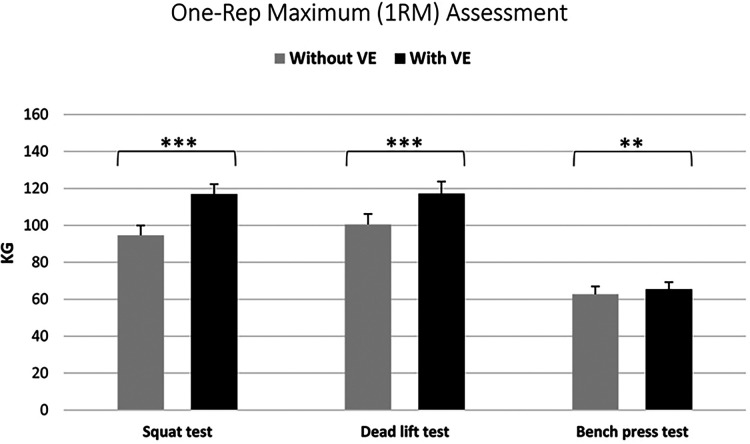
Comparison of strength performance in 1RM squat, deadlift, and bench press exercises without and with verbal encouragement (** significant difference at *p* < 0.01; *** significant difference at *p* < 0.001).

A Wilcoxon signed-rank test indicated a significant difference (Z = 3.13, *p* < 0.01) in the participants' 1RM deadlift performance results under normal conditions (median = 54.80, IQR = 42.13 kg) and VE (median = 58.44 kg, IQR = 44.08 kg). The results show that VE had a medium (Pearson's r = 0.45) positive effect on the participants' performance in the deadlift exercise (see Figure 1).

#### 3.1.2 Squat test

A paired samples *t*-test revealed a significant difference in the participants' 1RM squat test results under normal conditions (94.67 ± 36.6 kg) and VE (116.95 ± 37.14 kg), t(df = 47) = 9.49, *p* < 0.001. The results indicate that VE had a large positive effect (Hedges' *g* = 1.36) on the participants' performance in the 1RM squat test (see Figure 1).

#### 3.1.3 Deadlift test

A Wilcoxon signed-rank test uncovered a significant difference (Z = 5.44, *p* < 0.001) in the participants' 1RM deadlift performance results under normal conditions (median = 96.35 kg, IQR = 60.21 kg) and VE (median = 111.31 kg, IQR = 59.85 kg). The results indicate that VE had a large (Pearson's *r* = 0.79) positive effect on the participants' performance in the deadlift exercise (see Figure 1).

### 3.2 8-min time trials (8MTT)

**Figure 2:**
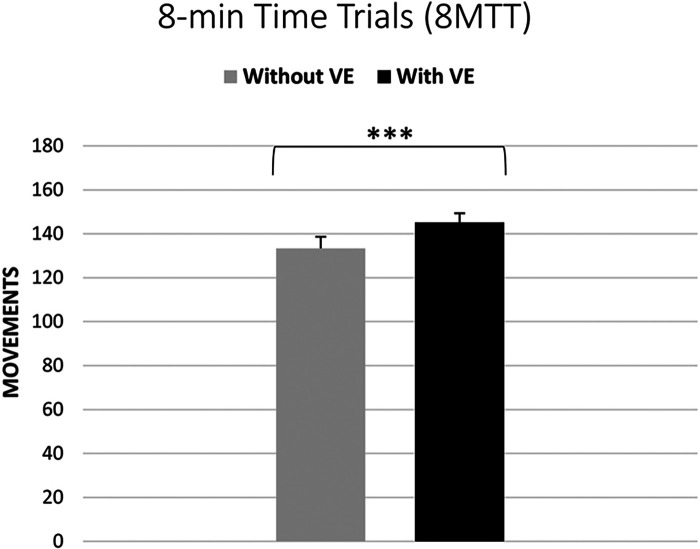
Comparison of endurance performance in the 8-min time trials without and with verbal encouragement (*** significant difference at *p* < 0.001).

The analysis using the Wilcoxon signed-rank test revealed a significant difference (*Z* = 6.04, *p* < 0.001) in the number of movements completed in the 8MTT under normal conditions (median = 130 movements, IQR = 34 movements) compared to the VE conditions (median = 138 movements, IQR = 35 movements). These findings suggest that VE had a large positive effect (Pearson's *r* = 0.87) on performance during the 8MTT, leading to an increased number of movements completed within the given time frame (see Figure 2).

### 3.3 Rating of perceived exertion (RPE)

**Figure 3:**
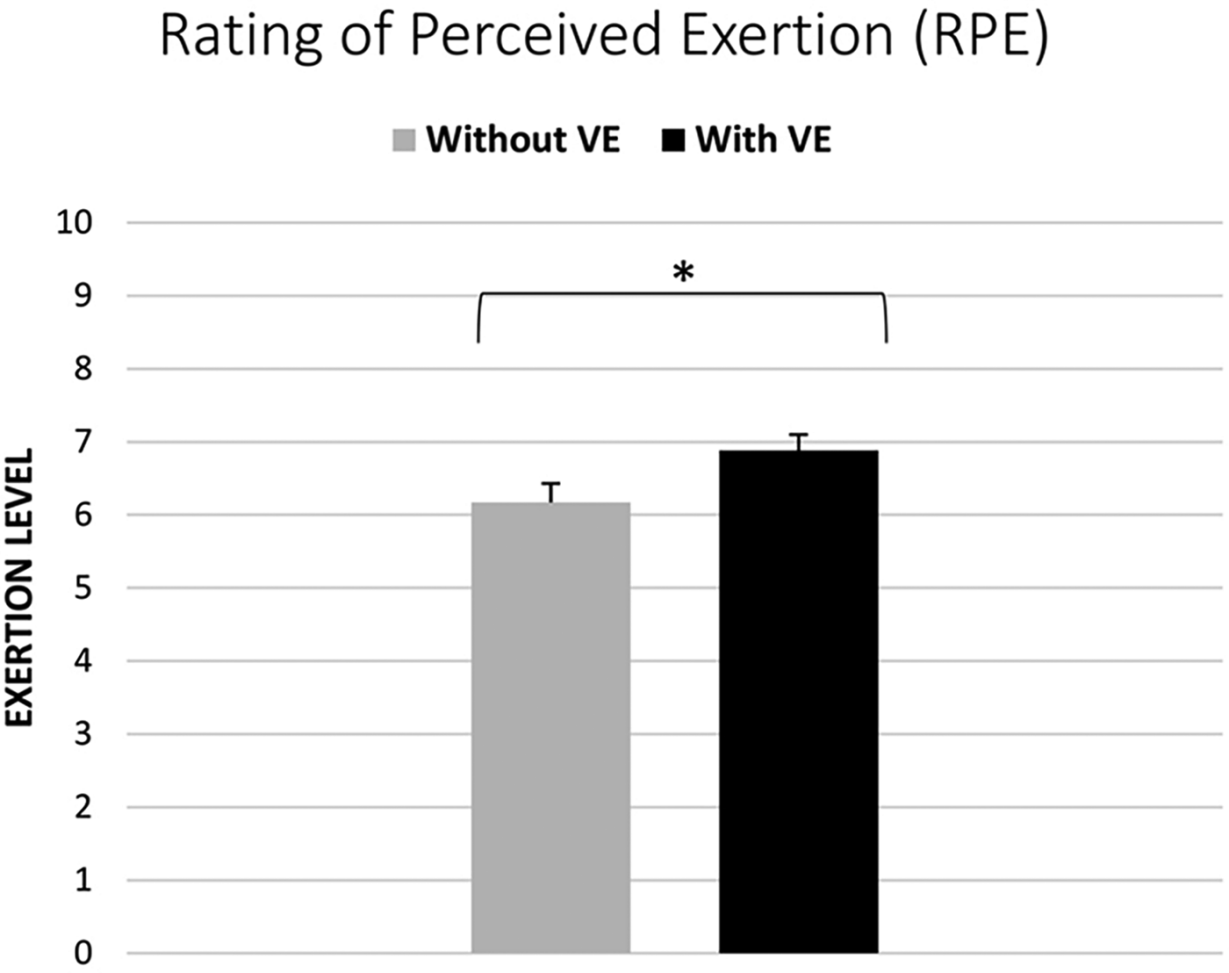
Perceived exertion levels under verbal encouragement (VE) vs. normal conditions: Rating of Perceived Exertion (RPE) analysis (* significant difference at *p* < 0.05).

Using the Wilcoxon signed-rank test, the findings indicate that participants reported higher levels of exertion under the condition of VE (median = 7, IQR = 2) compared to the normal condition (median = 6, IQR = 3). The difference in perceived exertion between the two conditions was found to be statistically significant (*Z* = 2.02, *p* < 0.05), albeit with a 0.04 significance level and a small effect size (Pearson's *r* = 0.29) (see Figure 3).

### 3.4 Physical activity enjoyment

**Figure 4:**
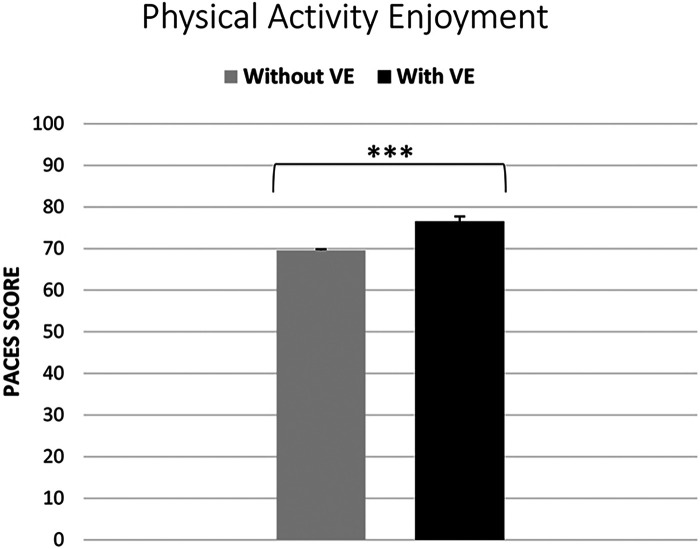
Perceived exertion levels under verbal encouragement (VE) vs. normal conditions: Rating of Perceived Exertion (RPE) analysis (***, significant difference at *p* < 0.001). The scores obtained from the PACES range between 18 and 126 and are derived from the sum of responses to 18 specific items.

A paired-samples Wilcoxon Signed Ranks Test was conducted to compare the physical activity enjoyment data collected with (median = 75, IQR = 8) and without VE (median = 69.5, IQR = 7). The results indicated a significant difference between the conditions, (Z = 5.61, *p* < 0.05) in favor of the VE conditions, with a large effect (Pearson's r = 0.81) (see Figure 4).

## 4 Discussion

The primary objective of this study was to investigate the effects of teacher verbal encouragement (VE) as a pedagogical tool on the strength and endurance performance of sports science students. Specifically, the study examined the impact of VE on the 1RM bench press, squat, and deadlift tests, as well as the 8-minute time trials (8MTT). The findings revealed that VE had a significant and beneficial effect on performance in all of these tests. The findings of the present study are in line with and supported by previous research conducted by McNair et al. (36), Kim and Kim (37), and Engel et al. (19). Although the populations and settings may differ between studies, the consistent pattern of results across different studies strengthens the validity and generalizability of the findings regarding the beneficial effects of VE on strength and endurance performances in the context of sports science education.

Expanding on existing literature, our findings underscore the importance of VE in education. Tuckman and Sexton (25) emphasize its role in boosting students' self-esteem and academic achievement. Similarly, Brown and Howard (26) demonstrate increased engagement in math learning with VE, while Guéguen et al. (27) show improved performance across tasks in children. These studies collectively emphasize the value of incorporating VE into teaching practices to enhance student outcomes. Our study reinforces the significant role of VE in improving student performance in sports science education, echoing findings from diverse educational contexts. Recognizing the efficacy of VE, educators can leverage verbal encouragement to optimize learning outcomes.

The observed improvements in strength performance under VE can be attributed to several factors. Firstly, VE has been shown to enhance motivation and self-efficacy, which are crucial psychological factors influencing performance outcomes (5). The positive and supportive words from the teacher likely boosted the students' confidence, belief in their abilities, and motivation to exert maximal effort during the strength tests. This increased motivation and self-efficacy likely translated into greater force production and improved performance in the 1RM tests. Furthermore, the teacher's VE may have played a role in directing the students' focus and attention during the strength tests. By providing specific cues and feedback, the teacher helped the students optimize their technique, maintain proper form, and generate maximum force during each lift (2, 38). The verbal cues and instructions from the teacher may have enhanced movement efficiency, muscle activation, and overall performance in the strength tests (2, 37).

In the case of the 8-minute time trials, the beneficial effects of VE can be attributed to its impact on endurance performance. VE has been shown to increase motivation, drive, and perseverance during high-intensity activities (12, 19). The encouraging words from the teacher likely provided an additional source of motivation and mental support for the students, enabling them to sustain a higher intensity and complete more repetitions or movements within the given time frame. The social aspect of VE should also be considered. The presence of a supportive teacher who provided personalized encouragement, addressed students by name, and created a positive and motivating environment likely fostered a sense of camaraderie and group cohesion. This social support can have a profound impact on endurance performance, as individuals tend to push harder and perform better in environments where they feel supported and connected (18, 39, 40).

The higher perceived exertion reported under VE suggests that the students perceived their physical effort to be greater when they received VE from the teacher. The verbal cues and motivational statements provided by the teacher may have increased the students' engagement and mental focus, leading them to exert more effort during the tests. The encouraging words may have instilled a sense of challenge and determination, prompting the students to push themselves harder and perceive their exertion levels as higher (8). On the other hand, the greater physical activity enjoyment reported under VE suggests that the students derived more pleasure and satisfaction from the exercises when they received VE. This latter can create a positive and motivating atmosphere that enhances the students' enjoyment of physical activity (8). The supportive and motivating words from the teacher may have fostered a sense of competence, autonomy, and connectedness among the students, thereby increasing their enjoyment of the exercises (5). One key aspect of this study is its consideration of subjective perceptions and enjoyment of physical activity. By prioritizing these aspects, sports science students are not only encouraged to perform but also to cultivate conducive learning environments conducive to effective teaching.

Taken together, the findings regarding perceived exertion and physical activity enjoyment suggest that VE plays a significant role in shaping the students' subjective experiences during exercise. The combination of higher perceived exertion and greater enjoyment under VE indicates that the students were not only exerting more effort but also finding the experience more enjoyable and rewarding. It is important to note that while the results demonstrate the positive effects of VE, the specific mechanisms underlying these effects warrant further investigation. Future research could explore factors such as the timing and frequency of VE, individual differences in response to encouragement, and the potential interaction between VE and other performance-enhancing strategies.

One limitation of this study is the restricted number of repetitions allowed in the 1RM tests due to time constraints. Participants were only permitted three repetitions, starting with the starting weight, which may have hindered their ability to achieve higher lifts and accurately determine their maximum strength capabilities. This limitation could have potentially underestimated the participants' true strength potential, compromising the overall conclusions drawn from the study. Another limitation is the use of self-reporting instruments to measure perceived exertion and physical activity enjoyment without undergoing a thorough cross-cultural validation process, particularly in Arab-speaking contexts. This oversight raises concerns about the instruments' reliability and validity in accurately capturing the intended constructs among Arab-speaking participants, limiting the generalizability of the study's findings in these populations.

## 5 Conclusions

The key findings of this study revealed significant improvements in strength and endurance performances when sports science students received verbal encouragement (VE) from their teacher. Participants demonstrated the ability to lift greater weights in the 1RM bench press, squat, and deadlift tests, indicating enhanced muscular strength. Moreover, they completed a greater number of repetitions in the 8-minute time trial (8MTT), reflecting improved endurance capacity under VE. Interestingly, participants reported lower perceived exertion during exercise performed under normal conditions, while physical activity enjoyment significantly increased with the presence of VE. These results suggest that the use of teacher VE in sports science education not only enhances strength and endurance training outcomes but also positively influences participants' psychological response to physical activity.

This study's findings offer practical implications for sport science educators, highlighting the role of communication in improving teaching efficacy. By emphasizing assertive communication strategies, such as VE, teachers in sports science education can create more engaging and supportive learning environments, thereby enhancing student physical engagement and performance. Looking forward, future research could explore specific communication techniques in greater depth and investigate the integration of technology-mediated communication tools. Such endeavors hold promise for further optimizing instructional practices and expanding learning opportunities within the field of sport sciences education.

## Data Availability

The data are not publicly available due to legal and ethical restrictions. The data presented in this study are available on request from the corresponding author.
